# Preoperative CT findings and prognosis of pulmonary sarcomatoid carcinoma: comparison with conventional NSCLC of similar tumor size

**DOI:** 10.1186/s12880-023-01065-8

**Published:** 2023-08-14

**Authors:** Wenjian Tang, Chunju Wen, Yixiu Pei, Zhen Wu, Junyuan Zhong, Jidong Peng, Jianping Zhong

**Affiliations:** 1 Department of Medical Imaging, Ganzhou People’s Hospital, The Affiliated Ganzhou Hospital of Nanchang University, 16th Meiguan Avenue, Ganzhou, 341000 P.R. China; 2 Department of Medical Hematology, Ganzhou People’s Hospital, The Affiliated Ganzhou Hospital of Nanchang University, Ganzhou, China; 3 Department of Pathology, Ganzhou People’s Hospital, The Affiliated Ganzhou Hospital of Nanchang University, Ganzhou, China

**Keywords:** Pulmonary sarcomatoid carcinoma, Non-small cell lung cancer, Computed tomography

## Abstract

**Background:**

Pulmonary sarcomatoid carcinoma (PSC) is a rare subtype of non-small cell lung cancer (NSCLC) but differs in terms of treatment strategies compared with conventional-NSCLC (c-NSCLC). However, preoperative CT differentiation between PSC and c-NSCLC remains a challenge. This study aimed to explore the CT findings and prognosis of PSC compared with c-NSCLC of similar tumor size.

**Methods:**

Clinical data and CT findings of 31 patients with PSC and 87 patients with c-NSCLC were retrospectively analyzed. Clinical data included sex, age, and smoking history. CT findings included tumor size, tumor location, calcification, vacuole/cavity, pleural invasion, mean CT value, and low-attenuation area (LAA) ratio. Kaplan‒Meier curves and log-rank tests were used for survival analysis. A Cox regression model was constructed to evaluate prognostic risk factors associated with overall survival (OS). The Spearman correlation among clinicoradiological outcomes were analyzed.

**Results:**

The mean tumor size of PSC and c-NSCLC were both 5.1 cm. The median survival times of PSC and c-NSCLC were 8 months and 34 months, respectively (*P* < 0.001). Calcification and vacuoles/cavities were rarely present in PSC. Pleural invasion occurred in both PSC and c-NSCLC (*P* = 0.285). The mean CT values of PSC and c-NSCLC on plain scan (PS), arterial phase (AP), and venous phase (VP) were 30.48 ± 1.59 vs. 36.25 ± 0.64 Hu (*P* = 0.002), 43.26 ± 2.96 vs. 58.71 ± 1.65 Hu (*P* < 0.001) and 50.26 ± 3.28 vs. 64.24 ± 1.86 Hu (*P* < 0.001), the AUCs were 0.685, 0.757 and 0.710, respectively. Compared to c-NSCLC, PSC had a larger LAA ratio, and the AUC was 0.802, with an optimal cutoff value of 20.6%, and the sensitivity and specificity were 0.645 and 0.862, respectively. Combined with the mean CT value and LAA ratio, AP + VP + LAA yielded the largest AUC of 0.826. The LAA ratio were not independent risk factors for PSC in this study. LAA ratio was negatively correlated with PS (r = -0.29), AP (r = -0.58), and VP (r = -0.66). LAA showed a weak positive correlation with tumor size(r = 0.27).

**Conclusions:**

PSC has a poorer prognosis than c-NSCLC of similar tumor size. The mean CT value and LAA ratio contributes to preoperative CT differentiation of PSC and c-NSCLC.

## Background

Non-small cell lung cancer (NSCLC) originates in epithelial tissue and has a variety of subtypes, accounting for approximately 85% of all lung cancers [1]. Pulmonary sarcomatoid carcinoma (PSC) is a rare subtype of NSCLC, accounting for approximately 1% of NSCLC, and is characterized by both epithelial and mesenchymal cells (> 10%) [2]. The presence of sarcomatous components suggests high-grade lesions that are highly invasive and metastatic, often leading to missed opportunities for surgical treatment [3–8].

The clinical management of PSC remains challenging. PSC patients are resistant to platinum-based chemotherapy regimens, which are effective for conventional-NSCLC (c-NSCLC) [9–11]. In addition, the common genetic mutations (e.g., ALK and EGFR) of c-NSCLC are rare in PSC [12–14]. Recent studies suggest that MET mutations and PD-L1 overexpression may be the key genetic events leading to the sarcomatoid transformation of c-NSCLC, which brings hope for molecular targeted therapy for PSC [14–16]. Therefore, it is important to distinguish PSC from c-NSCLC due to the differences in therapeutic strategies.

CT examination is the routine method to noninvasively detect tumor lesions of the lung. Although PSC with a biphasic histological component is well recognized in pathology [17], preoperative CT differentiation diagnosis between PSC and c-NSCLC remains a challenge. Previous reports found that PSC tumor lesions often appear as low-attenuation area (LAA) on CT images [18–20]. Whether LAA can be used as a basis for the differential diagnosis of PSC and c-NSCLC needs to be further explored.

The aim of this study was to retrospectively analyze the preoperative CT findings of PSC and c-NSCLC patients with similar tumor sizes. Moreover, the relationship between clinicoradiological outcomes and overall survival of PSC was further explored to improve the understanding of PSC.

## Methods

### Patients

A total of 118 patients, including 31 PSC and 87 c-NSCLC patients who underwent surgical resection (19 cases of PSC and all c-NSCLC) or needle biopsy (12 cases of PSC), were retrospectively studied in our hospital from January 2012 to December 2021. The inclusion criteria of c-NSCLC were as follows: (1) the diagnosis was made by light microscopic findings and immunohistochemistry after surgical resection; (2) tumor size > 2 cm; and (3) plain and enhanced CT scans acquired preoperatively. The exclusion criteria were as follows: (1) preoperative chemoradiotherapy performed at the time of CT examination; (2) poor image quality or missing images in the picture archiving and communication system (PACS); and (3) lost to follow-up. This study was approved by the institutional review board.

All available clinical data, including sex, age, and smoking history, were acquired by two of the authors (T.WJ, W.CJ). Tumors were classified and staged according to the eighth edition of the Tumor-Node-Metastasis (TNM) classification of NSCLC. Twelve PSC biopsy cases were clinically staged based on CT examination, while the 19 cases of PSC and all c-NSCLC patients were pathologically staged. The c-NSCLC included 49 cases of adenocarcinoma and 38 cases of squamous carcinoma.

### CT image acquisition

CT images were acquired using 64-slice SOMATON Definition AS (SIEMENS). CT scans were acquired at full inspiration. The contrast material iohexol (Beijing Beilu Pharmaceutical Co., Ltd.) was injected into the antecubital vein at a rate of 3.5 mL/s (1.2 mL/kg of body weight, less than 70 mL in total). The parameters of the CT scan were as follows: the tube voltage was 120 KV, the tube current was automatically adjusted, the matrix was 512 × 512, the field of view (FOV) was 403 mm×403 mm, and the reconstructed slice thickness was 1.25 mm. Plain scan (PS), arterial phase (AP) and venous phase (VP) images were obtained. AP and VP scans were acquired 25 and 60 s after the contrast injection, respectively.

### CT scan interpretation

**Fig. 1:**
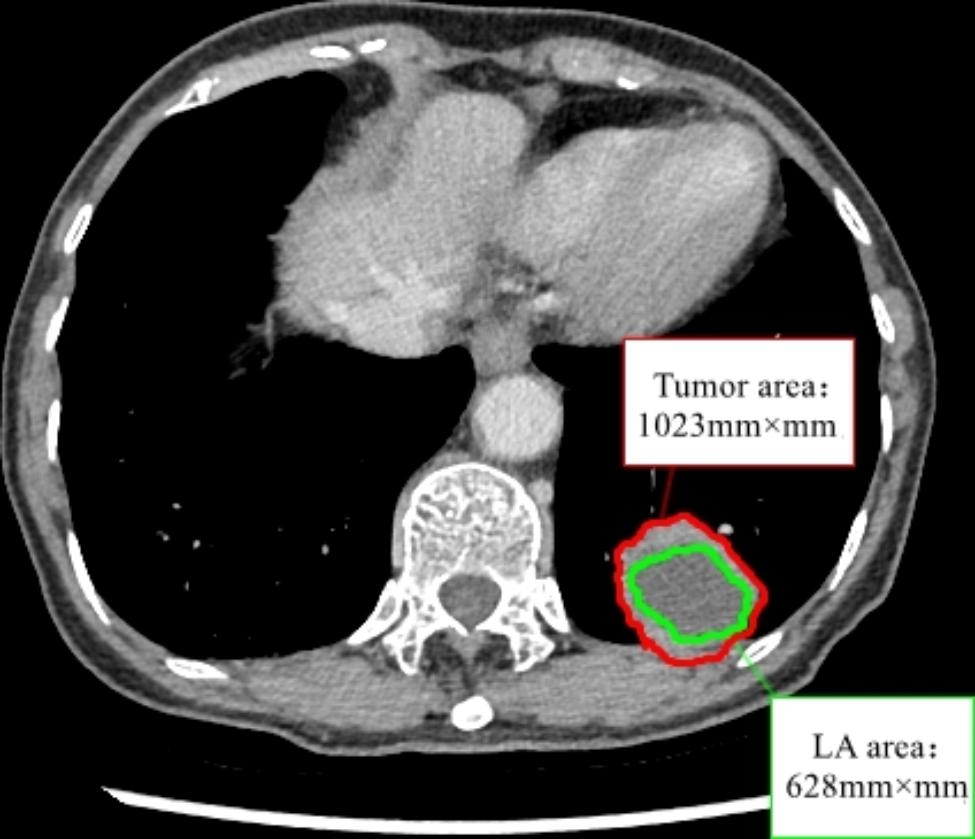
Measurement of the LAA ratio based on the VP image. The LAA ratio was defined as the percentage of the largest low-attenuation area (LAA) to the largest tumor area. In this case, the LAA ratio was 61%. This is the same case as in Fig. 2(**a**)-(**c**)

**Fig. 2:**
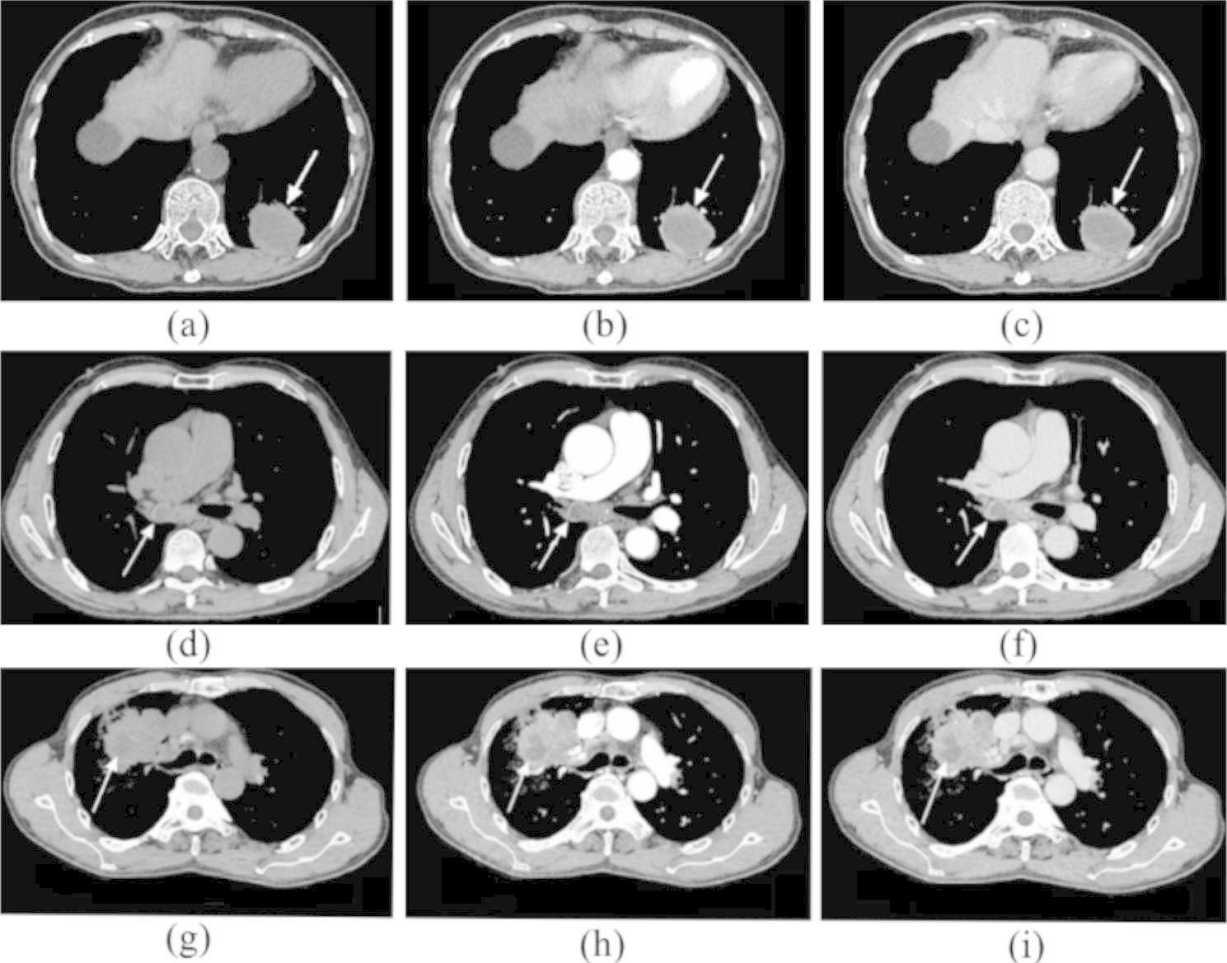
(**a**)-(**c**) A 68-year-old female with PSC in the left lower lobe (arrow). The LAA could be found at the central region of the lesion, with no enhancement on contrast-enhanced CT scans. In this case, the LAA ratio was 61%. (**d**)-(**f**) A 63-year-old male with PSC in the right lower lobe bronchus (arrow). Although the lesion is small, the LAA ratio is relatively large. The LAA ratio was 51%. (**g**)-(**i**) A 54-year-old male with adenocarcinoma in the right upper lobe. The LAA is seen within the tumor (arrow), but the LAA ratio is relatively small (19%)

In this study, three radiologists (T.WJ, P.YX and Z.JY) with more than 5 years of experience in using chest CT imaging for diagnosis retrospectively reviewed the CT images and evaluated the tumor size, tumor location, calcification, vacuole/cavity, pleural invasion, and LAA in the tumor. Mediastinal window images were used for analysis, WW 350 Hu and WL 40 Hu. The reconstruction kernel in mediastinal window was B31. Pleural invasion in surgical resection cases was determined by pathology, while 12 patients who underwent needle biopsy were considered to have pleural invasion if the boundary between the mass and visceral pleura was not clear on CT images. The maximum tumor layer was selected, and the mean CT value was measured in the PACS. Regions of interest (ROI) of the largest tumor layers and largest LAA layers were outlined in the VP sequence, and their areas were obtained. The LAA ratio was defined as the percentage of LAA to the tumor area (Fig. 1). If there was disagreement with the determination of the tumor boundary, further drawing was performed after discussion. Referring to previous studies [8, 19], LAA can be characterized by liquid density (from ­10 to 20 Hu) or equal density on plain scans with no enhancement on enhanced scans, while the peripheral solid component is significantly enhanced (Fig. 2).

An inter-observer agreement test of LAA was further conducted. Three radiologists first studied the LAA of PSC cases reported in the literature. According to the above LAA definition, three radiologists analyzed whether the CT images of PSC and c-NSCLC lesions had LAA. Three radiologists were blinded to the pathology results.

### Statistical analysis

Statistical analysis was performed with SPSS software (version 23.0) and Python (version 3.9.12). Data are expressed as the mean ± standard error. The clinical and CT findings were compared with *t* tests or Mann‒Whitney U tests, Pearson chi-square tests or Fisher exact tests. *Kendall’s W* test was used to assess inter-observer agreement of LAA. The median survival times of PSC and c-NSCLC patients were obtained with the *Kaplan‒Meier* method, and the differences between groups were analyzed by *log-rank* tests. Prognostic risk factors associated with OS were evaluated by a Cox regression model. Spearman correlation was used to explore the correlation among clinical data and CT findings of PSC. A *P* value less than 0.05 indicated a significant difference.

## Results

### Clinical and radiological features

Tables 1 and 2 display the clinical data and CT findings of PSC and c-NSCLC. There were 26 males and 5 females with PSC and 69 males and 18 females with c-NSCLC (*P* = 0.874). The mean tumor sizes of PSC (range 2.1–11.1 cm) and c-NSCLC (range 2.1–10.3 cm) were both 5.1 cm (*P* = 0.967). The age of PSC group (45–79 years, 62.81 ± 1.51) was older than that of c-NSCLC (38–83 years, 59.68 ± 0.98), *P* = 0.098. Most of the patients in the both groups were smokers (*P* = 0.484).

**Fig. 3:**
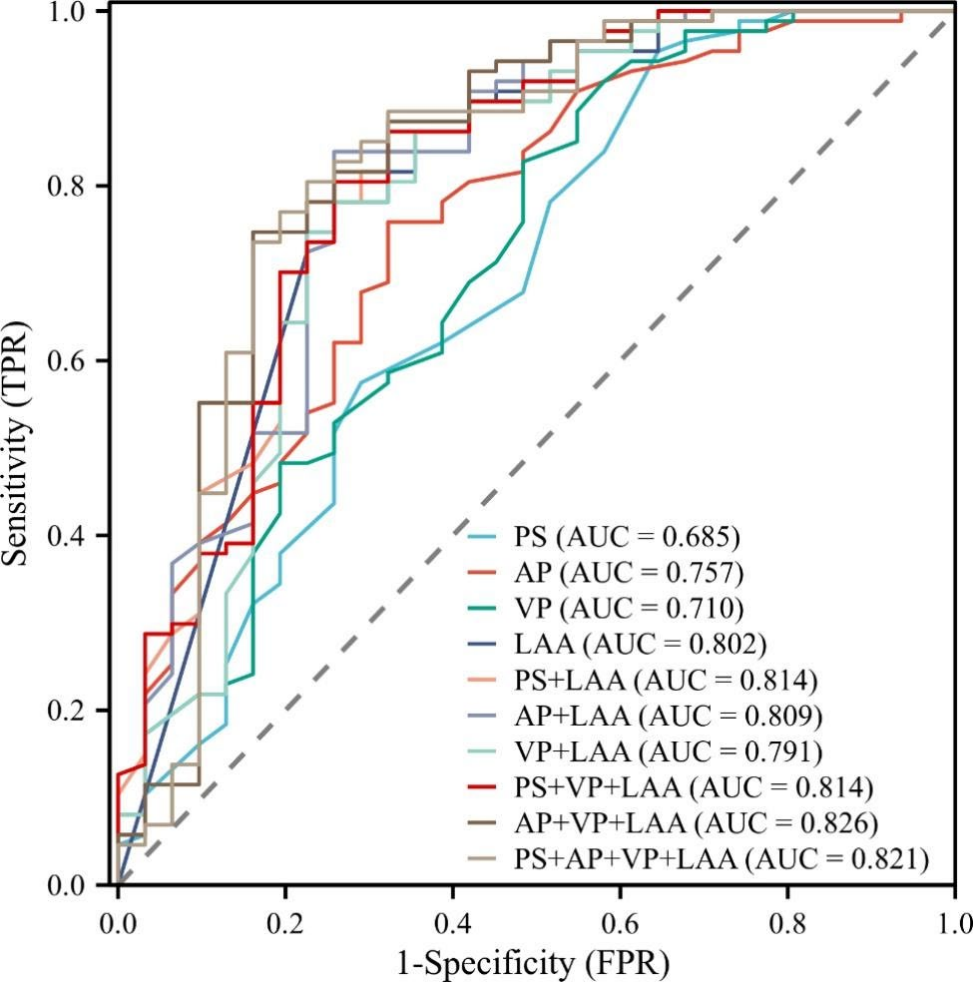
ROC curves of the mean CT value, LAA ratio and combined model in differentiating between PSC and c-NSCLC

In the PSC and c-NSCLC groups, 15 vs. 44 lesions occurred in the upper and middle lobes, while 16 vs. 43 lesions occurred in the lower lobes (*P* = 0.834). Calcification and vacuole/cavity (*P* = 0.056 and *P* = 0.072) were rarely present in the PSC group but could occur in c-NSCLC. Pleural invasion occurred in both groups (*P* = 0.285). On PS, the mean CT value of PSC was 30.48 ± 1.59 Hu, while that of c-NSCLC was 36.25 ± 0.64 Hu (*P* = 0.002), and the AUC was 0.685 (95% CI 0.568–0.802). On AP, the mean CT value of PSC and c-NSCLC was 43.26 ± 2.96 Hu vs. 58.71 ± 1.65 Hu (*P* < 0.001), and the AUC was 0.757 (95% CI 0.656–0.859). On the VP, the mean CT value of PSC and c-NSCLC was 50.26 ± 3.28 Hu vs. 64.24 ± 1.86 Hu (*P* < 0.001), and the AUC was 0.710 (95% CI 0.597–0.824). The LAA ratio of the tumors significantly differed between PSC (28.56 ± 3.83%) and c-NSCLC (6.28 ± 1.27%) (*P* < 0.001). Compared to c-NSCLC, PSC had a larger LAA ratio, the AUC was 0.802 (95% CI 0.701–0.903), with an optimal cutoff value of 20.6%, and the sensitivity and specificity were 0.645 and 0.862, respectively. Combined with the mean CT value and LAA ratio, AP + VP + LAA had the largest AUC of 0.826 (95% CI 0.726–0.925) (Fig. 3 in detail). The inter-observer agreement test of the LAA yielded Kendall’s W coefficient = 0.874 (*P* < 0.001).

**Table 1:** Clinical data and CT findings of PSC and c-NSCLC

Characteristic	PSC	c-NSCLC	*P*-value
*Gender*, n (%) Man Female	25 (80.6%) 6 (19.4%)	69 (79.3%) 18 (20.7%)	0.874
*Age* (year)	62.81 ± 1.51	59.68 ± 0.98	0.098
*Smoke*, n (%) No Yes	11 (35.5%) 20 (64.5%)	25 (28.7%) 62 (71.3%)	0.484
*Tumor size* (cm)	5.12 ± 0.38	5.11 ± 0.20	0.967
*Location*, n (%) Upper and middle Lower	15 (48.4%) 16 (51.6%)	44 (50.6%) 43 (49.4%)	0.834
*Calcification*, n (%) Absent Present	29 (93.5%) 2 (6.5%)	69 (79.3%) 18 (20.7%)	0.056
*Vacuole/cavity*, n (%) Absent Present	29 (93.5%) 2 (6.5%)	70 (80.5%) 17 (19.5%)	0.072
*Pleural invasion*, n (%) Absent Present	17 (54.8%) 14 (45.2%)	38 (43.7%) 49 (56.3%)	0.285
*Lymph node metastasis*, n (%) Absent Present	13 (41.9%) 18 (58.1%)	46(52.9%) 41(47.1%)	0.296
*Overall stage*, n (%) I-II III-IV	11 (35.5%) 20 (64.5%)	43 (49.4%) 44 (50.6%)	0.181
*Mean CT value [Hu]* Plain scan Arterial phase Venous phase	30.48 ± 1.59 43.26 ± 2.96 50.26 ± 3.28	36.25 ± 0.64 58.71 ± 1.65 64.24 ± 1.86	0.002* < 0.001** < 0.001**
*LAA*, n (%) Absent Present	7 (22.6%) 24 (77.4%)	63 (72.4%) 24 (27.6%)	< 0.001**
*LAA Ratio*	28.56 ± 3.83%	6.28 ± 1.27%	< 0.001**^#^

*: *P <* 0.01, **: *P <* 0.001, ^#^: Mann‒Whitney U test, n: number of patients

**Table 2:** Clinical data, CT findings and TNM stage of PSC

Case No.	Age (y)	Sex	Lobe	Location	Size (cm)	Calcifi- cation	Vacuole/ cavity	Pleural invasion	LAA ratio	TNM stage
1	54	M	Right upper	Peripheral	2.3	-	-	+	0	T2aN0M0 (IB)
2	66	M	Right upper	Peripheral	3.1	-	-	-	0.315	T2aN0M0 (IB)
3	56	M	Left main bronchus	Central	4.2	+	-	-	0	T2bN0M0 (IIA)
4	45	F	Right upper	Peripheral	4.8	-	-	-	0.06	T2bN0M0 (IIA)
5	67	M	Right upper	Peripheral	4.1	-	-	-	0.166	T2bN0M0 (IIA)
6	68	F	Left lower	Peripheral	4.1	-	-	-	0.614	T2bN0M0 (IIA)
7	68	M	Left lower	Peripheral	2.1	-	-	-	0	T1cN1M0 (IIB)
8	55	M	Left upper	Peripheral	3.1	-	-	-	0.368	T2aN1M0 (IIB)
9	72	M	Left lower	Peripheral	4.2	-	-	-	0.502	T2bN1M0 (IIB)
10	64	M	Left lower	Peripheral	5.2	-	-	+	0.508	T3N0M0 (IIB)
11	66	M	Left lower	Peripheral	6.9	-	+	-	0.514	T3N0M0 (IIB)
12	68	M	Right lower	Peripheral	3.6	-	-	+	0.211	T2aN2M0 (IIIA)
13	76	M	Right lower	Peripheral	2.8	-	-	-	0.446	T1cN2M0 (IIIA)
14	63	M	Right middle bronchus	Central	3.6	-	-	-	0.51	T2aN2M0 (IIIA)
15	79	M	Left upper	Peripheral	7.3	-	-	+	0.516	T4N0M0 (IIIA)
16	56	M	Right upper	Peripheral	4.8	-	+	+	0.617	T4N0M0 (IIIA) ^#^
17	46	F	Left lower	Peripheral	5.2	-	-	+	0	T3N2M0 (IIIB)
18	61	M	Left upper	Peripheral	7.1	-	-	+	0	T4N2M0 (IIIB)
19	63	M	Left lower	Peripheral	8.2	-	-	-	0.274	T4N2M0 (IIIB)
20	71	M	Left lower	Peripheral	11.1	+	-	+	0.298	T4N2M0 (IIIB)
21	59	M	Right lower	Peripheral	5.7	-	-	-	0.314	T3N2M0 (IIIB)
22	69	M	Left upper	Peripheral	7.9	-	-	+	0.362	T4N2M0 (IIIB)
23	56	M	Left lower	Peripheral	9.3	-	-	+	0.492	T4N2M0 (IIIB)
24	64	M	Left lower	Central	7.2	-	-	-	0.474	T4N3M0 (IIIC)
25	53	F	Right upper	Peripheral	4.1	-	-	-	0	T2bN0M1 (IV)
26	52	F	Right middle	Peripheral	5.3	-	-	-	0	T3N2M1 (IV)
27	64	M	Left upper	Peripheral	3.2	-	-	-	0.088	T2aN0M1 (IV)
28	56	M	Left upper	Peripheral	2.8	-	-	+	0.105	T2aN2M1 (IV)
29	72	F	Left upper	Peripheral	4.5	-	-	+	0.224	T2bN2M1 (IV)
30	74	M	Right lower	Peripheral	5.7	-	-	+	0.362	T3N1M1 (IV)
31	64	M	Left lower	Peripheral	5.3	-	-	+	0.514	T3N0M1 (IV)

Note: (+) = present, (-) = absent

^#^: this case has chest wall and paravertebral soft tissue invasion

### TNM classification

In PSC patients, the overall stage according to TNM classification was stage I in 2 patients (IB: 2), stage II in 9 patients (IIA: 4, IIB 5), stage III in 13 patients (IIIA: 5, IIIB: 7, IIIC: 1) and stage IV in 7 patients. The tumor status was T1 in two patients (T1c: 2), T2 in 14 patients (T2a: 7, T2b: 7), T3 in 7 patients and T4 in 8 patients. The nodal status was N0 in 13 patients, N1 in 4 patients, N2 in 13 patients, and N3 in one patient. The M status was M0 in 24 patients and M1 in 7 patients. There were 2 cases of brain metastasis, and the remaining 5 cases had metastasis to thoracic vertebrae, pleura, bilateral adrenal glands, mandible and clivus, respectively.

In c-NSCLC patients, overall stage I was observed in 9 patients (IA_3_:4, IB: 5), stage II in 34 patients (IIA: 14, IIB: 20), stage III in 43 patients (IIIA: 31, IIIB: 12) and stage IV in one patient. The tumor status was T1 in five patients (T1c: 5), T2 in 37 patients (T2a: 12, T2b: 25), T3 in 27 patients and T4 in 18 patients. The nodal status was N0 in 47 patients, N1 in 14 patients, and N2 in 26 patients. The M status was M0 in 86 patients and M1 in one patient, with metastasis to the thoracic spine.

#### Long-term survival

**Fig. 4:**
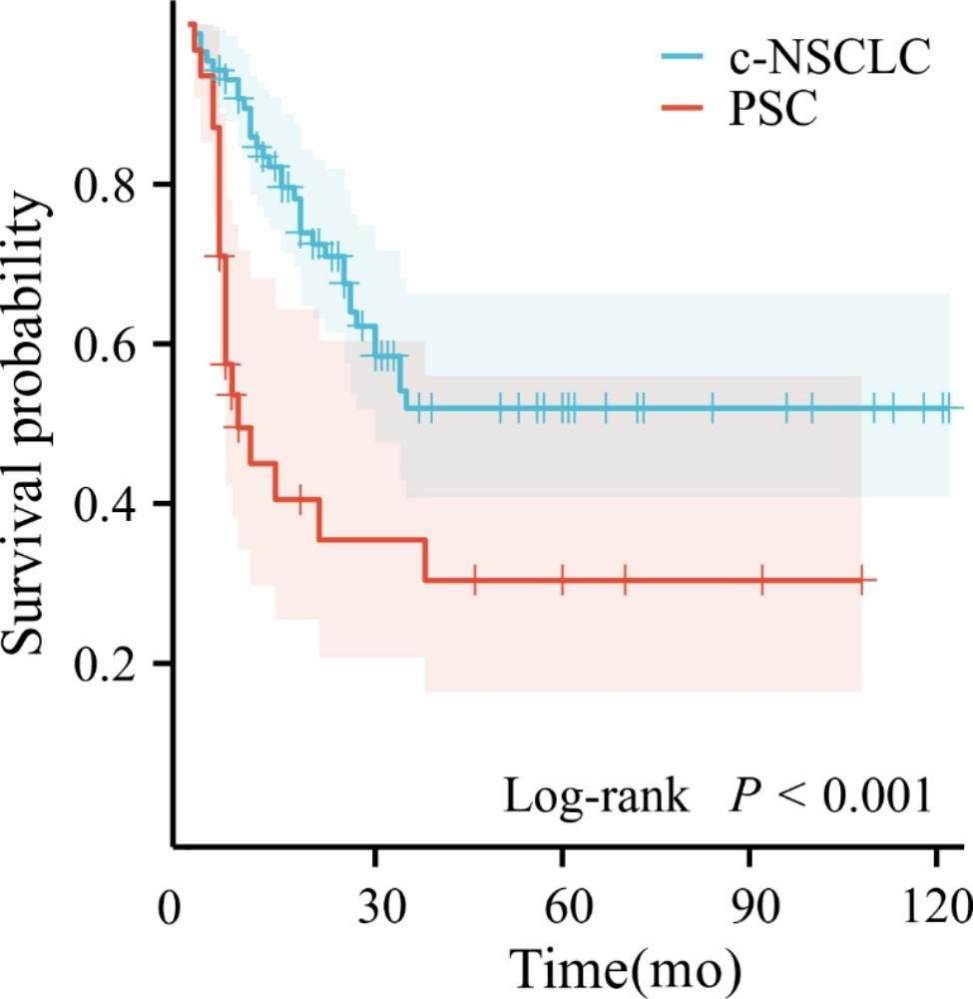
Kaplan‒Meier curves of PSC and c-NSCLC patients with similar tumor size

The median survival times of PSC and c-NSCLC were 8 months vs. 34 months (*P* < 0.001) (Fig. 4). The one-year and three-year overall survival (OS) rates in PSC and c-NSCLC patients were 32.3% vs. 79.3% and 19.4% vs. 27.6%, respectively.

Pleural invasion (*P* = 0.035), distant metastasis (*P* = 0.008), and overall stage (I-II vs. III-IV) (*P* = 0.002) were significantly associated with the overall survival of PSC patients, while LAA (*P* = 0.622), T status (T1-2 vs. T3-4) (*P* = 0.455), N status (N0 vs. N1-3) (*P* = 0.138), age (≥ 60 y vs. <60 y) (*P* = 0.656), and tumor size (≥ 5 cm vs. <5 cm) (*P* = 0.754) were not significantly associated with OS. The three parameters with significant differences (*P* < 0.05) were included in a multivariate Cox model. Only overall stage (I-II vs. III-IV) was found to be independently associated with OS (*P* = 0.009), HR = 5.37 (95% CI 1.53–18.82). Pleural invasion (*P* = 0.314) and distant metastasis (*P* = 0.181) were not independent risk factors for PSC.

### Correlation among clinicoradiological outcomes of PSC

**Fig. 5:**
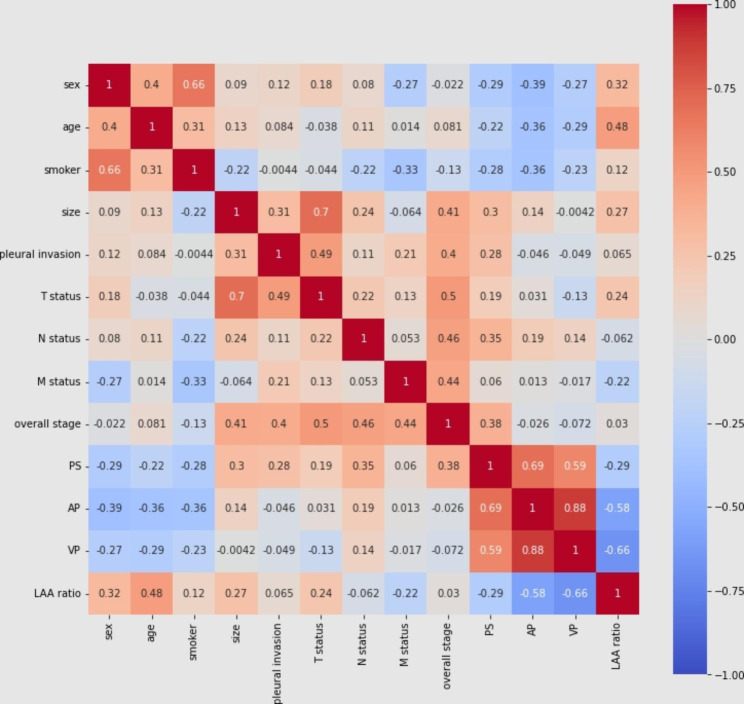
Correlation coefficients among clinical data and CT findings of PSC

The LAA ratio was negatively correlated with PS (r= -0.29), AP (r= -0.58), and VP (r= -0.66), but positively correlated with age (r = 0.48). LAA showed a weak positive correlation with tumor size(r = 0.27). Tumor size (r = 0.41), pleural invasion(r = 0.40), T status(r = 0.50), N status(r = 0.46) and M status(r = 0.44) were positively correlated with overall stage. Tumor size (r = 0.70) and pleural invasion(r = 0.49) were positively correlated with T status. Smoking status was positively correlated with sex (r = 0.66) (Fig. 5 in detail).

## Discussion

PSC is a rare subtype of NSCLC, and the latest 2021 WHO classification of lung neoplasms categorized PSC into three pathological subtypes: pleomorphic carcinoma (in the form of spindle and/or giant cells), carcinosarcoma, and pulmonary blastoma [21]. Although PSC with a biphasic histological component is well recognized in pathology [17], preoperative CT findings in differentiating between PSC and c-NSCLC remains a challenge.

In the present study, PSC patients were more likely to be male smokers, with a male-to-female ratio of 5.2:1. The average age at diagnosis was 63 years, and the average tumor size was 5.1 cm, which was similar to previous reports [4, 10, 19, 22]. Our study showed that the median survival time and the one-year and three-year survival rates of PSC patients were shorter than those of c-NSCLC patients with similar tumor size. This indicates that PSC is a special subtype of NSCLC that is associated with worse outcomes.

In this study, the CT findings showed that calcification and vacuole/cavity were rare in PSC, which is consistent with the previous study [18, 22]. However, Ung and colleagues [10] found that 20% of their cases had irregular cavities, which may be related to the small number of cases in our study. PSCs are prone to pleural invasion, and Kim and colleagues [8] showed that pleural invasion occurred in 7 out of 10 cases (70%). In the study of Fujisaki and colleagues [19], the pleural invasion rate was 43% (19/44). In our study, this rate was 45.2% (14/31), while c-NSCLC of similar sizes also had a high proportion of pleural invasion (36/56, 64.3%), suggesting that this CT finding is not specific—pleural invasion is not surprising in malignant tumors when the tumor is sufficiently large. However, pleural invasion is crucial for prognostic evaluation, and this study showed that pleural invasion was positively correlated with T stage and overall stage.

This study found that the most of PSC lesions presented with LAA (77.4%, 24/31), while LAA was absent in most of c-NSCLC lesions. In the study of Kim et al. [8], 80% (8/10) of PSC lesions had LAA. A similar study showed the presence of LAA in 90.9% (40/44) of PSC cases [18], which was consistent with our study. Our study showed that PSC had a larger LAA ratio than c-NSCLC, with an optimal cutoff value of 20.63%, and the sensitivity and specificity were 0.645 and 0.911, respectively. The pathological components of the LAA of PSC were mucinous degeneration, necrosis, and hemorrhage [8], suggesting that the rapid proliferation of tumor cells exceeded the blood supply [18]. In this study, LAA showed a weak positive correlation with tumor size, to some extent, consistent with the characteristics of hemorrhage and necrosis caused by rapid growth of PSC. However, the occurrence of LAA may be multifactorial, and the present study also found a positive correlation between LAA and age.

Such areas were found to be an independent predictor of PSC prognosis: patients with a larger LAA (> 25%) had a shorter overall/disease-free survival than those with a smaller LAA (**<** 25%) [19]. A study by Nishida et al. [18] yielded similar results. However, our results found that the LAA ratio was not associated with the prognosis of PSC. The reason was that PSC cases (7/31) in our study were prone to early distant metastasis, and there were 4 cases of T2 status and 3 cases of N0 status with distant metastasis. However, all PSC cases included in previous studies [18, 19] were surgical resection cases, and cases with early metastasis and missed opportunities for surgical treatment were not included. Even as a negative result, the finding that LAA was not significantly associated with survival could still provide valuable insights for future researchers.

Our study found that the mean CT value of PSC in plain and enhanced scans was lower than that in c-NSCLC. This is due to the high heterogeneity of PSC, and the tumor is often accompanied by necrosis. Our correlation analysis suggested a negative correlation between CT value and LAA, which was consistent with the discussion above—PSC had a lower mean CT value in plain and enhanced scans with a larger LAA ratio than c-NSCLC.

This study has several limitations. First, the number of PSC cases in this study was small, even though the cases dated back 10 years. Due to the rarity of PSC, only multicenter studies can guarantee a larger number of cases. Second, pulmonary abscess and tuberculosis are not considered in the differential diagnosis of PSC. However, sputum culture and blood culture can identify pathogenic bacteria, and the lesions obviously shrink after anti-inflammatory or anti-tuberculous therapy. Multicenter studies based on CT radiomics should further explore whether it is helpful for the preoperative diagnosis of PSC and predicting postoperative recurrence and overall survival time.

In conclusion, PSC has a worse prognosis than c-NSCLC of similar tumor size. Additionally, PSC had a lower mean CT value in plain and enhanced scans with a larger LAA ratio than c-NSCLC. These findings are helpful for the preoperative diagnosis of PSC and provide a reference for clinical treatment strategies.

## Data Availability

The datasets generated and/or analyzed during the current study are not publicly available as they contain identifiable and personal information but are available from the corresponding author on reasonable request.

## Abbreviations

PSC: Pulmonary sarcomatoid carcinoma
NSCLC: Non-small cell lung cancer
ALK: Anaplastic lymphoma kinase
EGFR: Epidermal growth factor receptor
PS: Plain scan
AP: Arterial phase
VP: Venous phase
LAA: Low-attenuation area
PACS: Picture archiving and communication system
AUC: Area under the curve
ROI: Region of interest
OS: Overall survival
CT: Confidence interval
